# Supplementary material for: Pigs and pasture: Drivers and characteristics of outdoor systems on the island of Ireland
Source: PLoS One. 2026 Feb 3;21(2):e0341440. doi: 10.1371/journal.pone.0341440 (PMC12867241; doi:10.1371/journal.pone.0341440)
Supplement: S1 Text — Questions annotated with * were the questions that had to be answered to include the participation in the study. (PDF) [file pone.0341440.s001.pdf]

S1 Text. Overall questionnaire. Questions annotated with * were the questions that had to be answered to include the participation in the study.

General farm information:

1.* In which county is your farm located? (Single choice, n=90)

- Antrim: 2
- Armagh: 0
- Carlow: 0
- Cavan: 3
- Clare: 6
- Cork: 10
- Donegal: 0
- Down: 2
- Dublin: 2
- Fermanagh: 0
- Galway: 5
- Kerry: 2
- Kildare: 8
- Kilkenny: 2
- Laois: 6
- Leitrim: 1
- Limerick: 1
- Londonderry/Derry: 0
- Longford: 0
- Louth: 2
- Mayo: 1
- Meath: 5
- Monaghan: 1
- Offaly: 1
- Roscommon: 0
- Sligo: 8
- Tipperary: 7
- Tyrone: 0
- Waterford: 4
- Westmeath: 1
- Wexford: 4
- Wicklow: 6

2.* Why do you keep pigs? (Multiple choice, n=90)

- Personal meat consumption: 59
- Selling pig meat: 49
- Breeding pigs for sale (all kind of pigs, not for pedigree): 35
- Land management (e.g. to turn over soil): 30
- Conservation of traditional, Irish and/or rare breed(s): 29
- Pet/hobby: 23
- Tourism (e.g. open farm): 9
- Showing pigs: 9
- Rescue sanctuary: 2
- Other (please specify): 4
    - “Pedigree breed”, “high welfare”, “for clean healthy chemical and GMO free food”, “help break life cycle of insect pest on Eucalyptus plantations”

3. What percentage of meat do you sell at each location below? *Numbers need to add up to 100%* (Multiple choice, n= 44, if "selling pig meat" selected in Q.2)

	Minimum	Median	Maximum	Average
Directly from the farm	0	50	100	53.95
Farmers market	0	0	100	12
Butchers shop	0	0	100	12.56
Supermarket chain	0	0	70	2.79
Online shop	0	0	100	11.37
Other	0	0	100	9.65

4. Where do you slaughter your pigs? (Multiple choice, n=49, if "selling pig meat" selected in Q.2)

- At a licenced slaughterhouse: 41
- At the butcher/victualler: 10
- Prefer not to say: 0
- Other (please specify): 2
    - Farmers had not reached point of slaughter yet (n=2)

5. Why do you slaughter your pigs there? (Multiple choice, n=46, if "At a licenced slaughterhouse " and/or "At the butcher/victualler" selected in Q.4)

- Closest location to my farm: 25
- They allow me to slaughter a small number of pigs: 21
- Only place that will accepts my pigs: 7
- Best slaughter rate: 3
- Other (please specify): 14
    - Human handling/high animal welfare (n=4), Organic certified abattoir (n=4), small abattoir/family business (n=2), abattoir used by/deliver to the butcher (n=2), abattoir that return the carcass to the farm (n=1), "quality of the butcher" (n=1), "Required by "Health Service Executive" HSE (n=1)

6. Do you have a herd number? (Single choice, n=74)

- Yes: 69
- No: 2
- Prefer not to say: 3

7.* Do you produce your pigs to meet a specific standard? (Single choice, n=90)

- No: 45
- Organic standard: 35
- GM free feed: 10

8.* How many people are involved in taking care of your pigs on a daily basis? (Open question, n=90)

- 1: 50
- 2: 27
- 3: 4
- 4: 4
- 5: 2
- 10: 2
- 15:1

9.* When did you start keeping pigs outdoors? (Single choice, n=90)

- 1992: 2
- 1995: 1
- 1997: 2
- 2000: 1
- 2003: 1
- 2005: 1
- 2006: 1
- 2007: 1
- 2008: 3
- 2009: 2
- 2010: 7
- 2011: 3
- 2010: 7
- 2013: 1
- 2014: 2
- 2010: 7
- 2016: 9
- 2017: 1
- 2018: 9
- 2019: 6
- 2020: 8
- 2021: 4
- 2022: 11

10.* Are you a member of a pig society? (Single choice, n=90)

- Yes: 47
- No: 40
- Prefer not to say: 3

11. What society/ies are you a member of? (Open question, n=45, if “yes” to Q.12)

- The Irish Pig Society: 37
- British Pig Association: 4
- British Kune-kune Pig Society: 3
- Oxford Sandy and Black society: 2
- Middle white society: 1
- Gloucestershire old spot breeders club: 1
- British Lop pig society: 1
- British Saddleback society: 1
- Berkshire pig society: 1

Husbandry:

12. At any one time, what is the maximum number of pigs you keep? *Please enter '0' if you don't have this type of animal* (Open question, n=88)

	Minimum	Median	Maximum	Average
Sow/Gilts	0	4	60	6.38
Boars	0	1	6	1.27
Grower pigs (less than 6 months old)	0	10	150	20.92
Piglets / unweaned pigs	0	12	100	16.94

13.* Where do you normally source your animals? (Multiple choice, n=89)

- Bred on your farm: 51
- From outdoor farm(s) from your personal/working network: 42
- From farm(s) from internet (DoneDeal or others), newspaper advert: 20
- From indoor farm(s) from your personal/working network: 11
- Other (Please specify): 4
    - “Rescues”, “UK”, “every imaginable scenario”, “three organic and local farms”

14. What breed are your pigs? (Multiple choice, n=90)

- Berkshire: 12
- British lop: 1
- British Saddleback: 12
- Duroc: 30
- Gloucester old spot: 18
- Hampshire: 6
- Kune kune: 14
- Landrace: 13
- Large black: 7
- Large white: 11
- Mangalitsa: 3
- Micropig: 2
- Middle white: 6
- Oxford sandy and Black: 30
- Piétran: 3
- Tamworth: 22
- Vietnamese Pot-bellied: 6
- Welsh: 2
- I don’t know: 2
- Other (please specify): 16
    - “Idaho pasture pigs” (n=1), “wild boar” (n=1), “Iron age pigs” (n=1), “Danbred2 (n=1), Cross breed (n=12)

15. What are the breed(s) of your sows, boars and grower pigs? (Multiple choice, n=89, only the breeds selected in Q. 8 appeared in this question)

	Sows/Gilts	Boars	Grower pigs (less than 6-7 months old)
Berkshire	8	4	8
British lop	1	0	0
British Saddleback	10	2	4
Duroc	9	13	21
Gloucester old spot	11	3	13
Hampshire	4	0	3
Kune kune	10	7	5
Landrace	7	2	7
Large white	5	2	6
Large black	6	1	2
Mangalista	3	1	1
Micropig	1	1	0
Middle white	4	4	1
Oxford Sandy and Black	22	4	23
Piétrain	2	2	1
Tamworth	15	8	14
Vietnamese Pot-bellied	5	2	0
Welsh	2	0	1
I do not know	1	0	1
[Name of the breeds written in the previous question]	7	3	12

16. What is the maximum total size of the outdoor area used for pigs? (Open question, n=84)

	Minimum	Median	Maximum	Average
Square meters	16	10235	3237000	60520.32

17. What is the land-type of the areas where you keep your pigs? (Multiple choice, n=87)

- Clay: 67
- Loam: 29
- Sand: 9
- Silt: 8

18. What is the nature of the underfoot surface in the outdoor area where you keep your pigs? (Multiple choice, n=88)

- Field/paddock (with vegetation): 72
- Scrubland (with bushes and scrubs): 33
- Woodland/forestry (with trees): 29
- Additional straw: 18
- Mucky contained area: 13
- Solid concrete: 8
- Tillage soil: 6
- Concrete slats: 2
- Other (Feel free to describe this for us): 2
  - Woodchip (n=2), rubber mat (n=1)

19. Do your animals have access to shade/shelter/an indoor area? (Multiple choice, n=87)

- Yes, mobile huts: 53
- Yes, building/barn opened 24h/7: 35
- Yes, natural shelter: 33
- Yes, fixed huts: 22
- Yes, building/barn opened at specific times: 10
- No: 0
- Other (Feel free to describe this for us): 0

Feed and water management:

20. How do you manage the feeding of your pigs? (Multiple choice, n=86)

- Pigs are fed outdoors and feeders are moved regularly to new locations within the outdoor area: 42
- Pigs are scatter fed outdoors: 33
- Pigs are fed outdoors and feeders are in specific fixed locations in the outdoor area: 16
- Pigs are fed outdoors from a single feeder: 12
- Pigs are fed inside from several feeders located in separate areas: 9
- Pigs are fed inside from a single feeder: 3

21. Do you adapt your feeding strategy based on the season (i.e. according to the animals' stage of production and /or food availability)? (Single choice, n=85)

- No: 31
- Yes, feel free to describe: 54
  - Feeding strategies based on the animals: age or growth stage (n=9), needs (n=1), body condition (n=1), mobility (n=1), extra feeding for sows in gestation or lactation (n=8), specific ration for sows in lactation (n=1)
  - Feeding strategies based forage, home-grown cereals or windfall fruits availability: pigs fed with fresh home-grown and seasonal vegetable, cereals and grass (n=5), less feeding in summer (n=3), less feeding when introduce to a new paddock (n=1), extra feeding when natural availability is running out (n=1),
  - Feeding strategies based on the weather(n=2), with extra feed in winter (n=7)
  - Animals brought and fed indoor during winter (n=4), more scatter feeding in summer (n=1)
  - Feeding labour: Feed is weighted (n=1), Creep feeder attached to the mobile shelter (n=1), manual feeding with buckets (n=1), piglets fed from a single large feeder but older animals fed from several feeder (n=1)

22. Where do you source your pig feed? *More detailed options in the next questions* (Multiple choice, n=86)

- Buy it from a feed merchant (e.g. feed-mill, co-op etc.): 76
- Home-grown: 19
- Other: 9
  - Whey from local cheese maker (n=2); brewer spent grain (n=1); organic or not tillage (n=2), organic grain (n=1) and, potatoes and beet (n=1) from local farmer, fruit and vegetables wholes sales excess (n=1), straight feeds (n=1), waste oats fermented at the farm (n=1),

23. Which feed is home-grown? *"1" being the most important component, cell can be left empty if your pigs are not fed with one of these options* (Ranking question, n=19, if “Home-grown” selected in Q.21)

Rank	1	2	3	4	5	6	7	8	9
Pasture / Grass	7	5	3	0	1	1	0	0	0
Vegetables	3	2	3	2	1	2	1	0	0
Barley	2	2	2	0	0	0	1	2	0
Silage	2	2	1	1	3	2	0	0	0
Hay	1	1	2	4	2	2	0	0	1
Wheat	1	2	1	0	1	0	0	1	3
Oat	1	0	3	4	1	0	1	2	2
Fruit	0	2	1	1	0	2	0	1	0
Corn	0	1	0	0	2	1	2	2	3
Haylage	0	0	1	3	3	3	3	0	0
Alfafa	0	0	0	1	1	2	4	3	1
Sorghum	0	0	0	0	0	0	0	0	1
Soybean	0	0	0	0	0	0	0	0	0
Other	1	2	0	1	1	0	0	0	1

24. Which kind of food do you buy from the feed merchant? *"1" being the most important component, cell can be left empty if your pigs are not fed with one of these options* (Ranking question, n=79, if “Buy it from a feed merchant” selected in Q.21)

Rank	1	2	3	4
Pellet	36	12	8	6
Meal	23	15	14	8
Fodder	14	14	16	4
Other	4	8	7	27

25. What other kinds of feed do you provide to your pigs? (Ranking question, n=73, if “Other” to Q.21)

Rank	1	2	3	4	5
Fruits and/or vegetables	47	9	6	3	1
Milk or milk by-products	6	10	10	11	3
Food waste	5	16	8	3	12
Feedstuff for other animals	2	7	11	10	11
Other	5	6	4	11	13

26. How do you manage your water supply to the pigs? (Multiple choice, n=84)

- Pigs have access to a single water location in the outdoor area: 32
- Pigs have access to water outdoors and the water areas are in specific fixed locations: 30
- Pigs have access to water outdoors and the water areas are moved regularly to new locations within the outdoor area(s): 22
- Pigs have access to several water locations in the inside area(s): 18
- Pigs have access to a single water location in the inside area: 12

27. What is the main drinking system? (Multiple choice, n=84)

- Trough automatically filled with running water: 50
- Trough manually filled with running water: 26
- Nipple drinkers connected to running water: 13
- Direct access to natural water supply (river, lake...): 7
- Trough manually filled with still water, lake, river water located next to the farm: 5
- Nipple drinkers connected to still water supply (e.g. a rainwater harvesting): 3
- Trough automatically filled with still water, lake, river water located next to the farm: 2

Animal health management:

28. Which of the following conditions have you observed on your farm in the last 12 months? (Multiple choice, n=83)

- None of the above: 57
- Heat stress: 9
- Lameness: 8
- Overweight: 4
- Reproductive/farrowing difficulties (boars and/or sows): 4
- Skin, tail and/or ear lesions: 4
- Cold stress: 3
- Gastrointestinal disorders (diarrhea): 3
- Respiratory symptoms: 3
- Poor body condition: 2
- Dehydration: 1
- Urinary issues: 1
- Watery eyes: 1
- High mortality in piglets: 0
- Adult mortality due to health issues (i.e. not due to old age) : 0
- Other: 0

29. In which class of pigs did the following diseases and/or parasitism occur in the last 12 months? (Multiple choice, n=42)

	Pigets / unweaned pigs	Weaned / adults pigs	Total
Actinobacillus pleuropneumonia (APP)	1	2	3
Dysentery (Brachyspira, Serpuline or Treponema)	2	1	3
Erysipelas	1	3	4
Escherichia coli	2	1	3
Ileitis (Lawsonia intracellularis)	1	1	2
Influenza	1	1	2
Mycoplasma hyopneumoniae	1	1	2
Parvovirus	1	1	2
Porcine circovirus 2 (PCV2)	1	1	2
Porcine respiratory and reproductive syndrome (PRRS)	1	1	2
Rhinitis	1	1	2
Salmonella	1	1	2
Streptococcus	1	1	2
Tetanus	2	1	3
Trichinosis	1	1	2
Skin parasites	2	10	12
Intestinal parasites	4	9	13
I do not know	6	7	13
Other[1] (please describe)	na	na	13

[1]: None of the above or nothing
na: not applicable

30. Which of the following do you use to treat and prevent diseases, parasitism and/or lesions in your animals? (Multiple choice, n=76)

- Good animal care (e.g. diet, comfort, space, shelter…): 71
- Good hygiene and equipment maintenance: 49
- Paddock rotation: 48
- Anthelmintics (worm and fluke treatments): 19
- Antibiotics: 15
- Quarantine: 13
- Vaccines: 11
- Homeopathy: 8
- Plants: 8
- Pain relievers: 6
- Antiseptics: 5
- Antivirals: 0
- Other: 0

31. If you need advice about your pigs' health and/or welfare, do you consult: (Single choice, n=85)

- A single vet: 44
- Farmers that raise pigs outdoor: 25
- Several veterinarians/veterinary practices: 7
- Prefer not to say: 1
- Farmers that raise pigs indoor: 0
- Other (Please specify): 8
  - The Irish pig society (n=2), I am a vet (n=2), single vet and farmers that raised pigs outside (n=2), All the options (n=1), did not contact a vet yet (n=1)

32. What kind of veterinarian do you consult? (Single choice, n=51, if "A single vet" or "Several veterinarians/veterinary practices" selected in Q.30)

- General farm/large animal specialist: 29
- Small and large animals specialist: 13
- Specialist with strong experience/knowledge of outdoor pig production: 11
- Specialist with strong experience/knowledge of conventional pig production: 4
- Small animal specialist: 1
- Other (please specify): 0

Farmers' attitudes and perception of the outdoor pig industry

33. Please tell us briefly what got you into outdoor pig raising in the first place? (Open question, n=67)

Answers were categorised in 9 groups related to:

1. Meat at 44.8% (n=30): participants raised concerns about indoor/conventional systems meat products and/or express the benefits of outdoor systems on the meat products. The characteristics of the meat cited by the participants were "taste", "quality", "medication" or "antibiotics", "vaccines", "nitrate and nitrite", "natural and clean", "food safety and security". Meat produced is for "own consumption"/"family"/"self-sufficiency" and/or the "local community". Meat from outdoor pig systems is considered as a "niche product" "rewarding […] to produce"; in which people "are interested" in the living area of the farmers, and that fill "a gap in the market".

2. Animal welfare at 37.3% (n=25): participants raised "concerns about indoor/conventional systems", and/or express the benefits of outdoor systems on the pigs' welfare. The characteristics of the animal welfare cited by the participants were "unethical", "excessive use of antibiotics", "practice that [farmer] does not agree with", "intensive" condition when describing conventional systems. The characteristics of the animal welfare cited by the participants were to give access to the pigs to "their natural environment" where they can "root, exercise" with the aim of offering pigs a "good quality of life" when describing outdoor systems.

3. Land management at 16.4% (n=11): participants expressed their interest in using pigs for "manure", "soil turnover", "weed control" and "clear woodland/forest", pigs being "the only animals compatible with agroforestry".

4. Love of the animal at 10.4% (n=7): participants expressed the "sentimental connection" they directly or a member of their family have with pigs.

5. Purposes at 10.4% (n=7): participants raised pigs outdoor different purposes such as "tourism", "hobby", "rescuing"

6. Breed conservation at 7.55 (n=5): participants expressed their interest for breed conservation being a "passion" or for "tourism" purpose.

7. Sustainability at 3% (n=2): participants associated outdoor systems to sustainability including "food sustainability".

8. Inheritance at 3% (n=2): participants inherited pigs from a "farmer leaving the sector" or the "family".

9. Other at 10.4% (n=7): participants raised pigs outdoor for "novelty" or "curiosity", for having the "space", or "access to food waste".

34. Please rate how important the following factors are to you. *1= not at all important, 5= extremely important*. I produce pigs this way because… (Single choice, n=75)

	Not at all important	Not important	Neither important nor unimportant	Important	Extremely important	No opinion/Not applicable
… I believe it offers better animal welfare (n=73)						
Sale	0	0	0	3	38	0
Other	0	0	1	2	29	0
Total	0	0	1	5	67	0
… I have concerns about conventional pig production (n=75)						
Sale	0	3	2	7	30	0
Other	2	1	3	6	21	0
Total	2	4	5	13	51	0
… I want my pigs to access the 5 freedoms of animal welfare * (n=75)						
Sale	0	1	0	4	37	0
Other	0	0	0	3	30	0
Total	0	1	0	7	67	0
… I see that there is an opportunity in the market for pigmeat from outdoor systems (n=75)						
Sale	1	2	1	18	20	0
Other	6	1	3	14	4	5
Total	7	3	4	32	24	5
… it is safer, tastier and better quality meat (n=75)						
Sale	0	0	1	6	35	0
Other	1	0	0	6	26	0
Total	1	0	1	1	61	0
… food provenance is important to me (n=75)						
Sale	0	0	1	12	29	0
Other	1	0	0	10	21)	1
Total	1	0	1	22	50	1

*: free from hunger and thirst; discomfort; pain, injury or disease; free to express normal behaviour; free from fear and distress

34bis. Please rate how important the following factors are to you. *1= not at all important, 5= extremely important*. I produce pigs this way because… (Single choice, n=75)

	Not at all important	Not important	Neither important nor unimportant	Important	Extremely important	No opinion/Not applicable
… they make an important contribution to my farm (soil health, they eat organic waste etc.) (n=75)						
Sale	0	2	7	15	18	0
Other	2	4	3	10	13	1
Total	2	6	10	25	31	1
… I believe it is more environmentally sustainable (n=75)						
Sale	0	0	2	16	24	0
Other	1	0	2	10	20	0
Total	1	0	4	26	44	0
… it reduces the feeding, buildings' maintenance cost (n=75)						
Sale	5	7	7	11	11	1
Other	2	7	9	6	7	2
Total	7	14	16	17	18	3
Other[1] (n=18)	1	0	4	2	4	7

[1]: "Important" that raising pig outdoor "fits within the natural cycle of my farm" (n=1), "Extremely important" that it "helps with pest control in our eucalyptus plantations" (n=1), No rating of "Pig production is still very expensive even when raised outdoors and it is hard to turn a profit when raising outdoor pigs on organic feed" (n=1)

35. What are the three main daily challenges of keeping pigs outdoors on your farm? (Open question, n=66, responses=174)

Codes:

- Feed: 35
- Fencing: 33
- Weather: 31
- Costs: 20
- Labour/Time: 16
- Paddock management: 16
- Animal management: 15
- Soil quality: 10
- Water: 9
- Infrastructure: 8
- Health: 7
- Maintenance: 7
- Bedding: 5
- Knowledge support: 3
- Land base: 3
- Pests: 2
- Slaughter/Processing: 2
- Enrichment: 1
- Market: 1
- Regulation: 1

Weather explainers:

- Wet/Rain: 5
- Dry: 3
- Cold: 3
- Sun: 3
- Winter: 2

Paddock management explainer:

- Rotation: 7

36. What are three main changes that are needed at infrastructural level (policy, education, advisory etc.) to encourage the production of pigs outdoors? (Open question, n=60, responses=151)

Codes:

- Education: 54
- Knowledge sharing: 25
- Financial support: 19
- Regulatory body: 12
- Marketing: 12
- Policy: 11
- Slaughter facilities: 6
- Premiums: 5
- Assurance: 4
- Support: 4
- High Welfare: 2
- Feed: 1
- Health: 1
- Land base: 1
- Motivation: 1

Educations explainers:

- Consumer: 13
- Producer: 4
- Welfare: 13
- Environment: 3

Knowledge sharing explainers:

- Advisory: 8
- Peer-to-peer: 7
- Vet: 1

Financial support explainers:

- Infrastructure: 4
- Feed: 1

37. How would you describe your farm? (Open question, n=66, responses=170)

Codes:

- Enjoyable: 17
- Small: 16
- High Welfare: 13
- Mixed:12
- Biodiverse: 10
- Non intensive: 9
- Organic: 8
- Smallholding: 7
- Sustainable: 7
- Traditional: 6
- Family: 5
- Hobby: 5
- Natural: 5
- Healthy: 4
- Non profitable: 4
- Regenerative: 4
- Educational: 3
- Clean: 2
- Community: 2
- Ethical: 2
- Profitable: 2
- Progressive: 2
- Scenic: 2
- Struggling: 2
- Balanced: 1
- Chemical free: 1
- Commercial: 1
- Efficient: 1
- Enriched: 1
- Farm: 1
- Lifestyle: 1
- Mucky: 1
- Needs work: 1
- Noisy: 1
- Not profitable: 1
- Pork: 1
- Remote: 1
- Rural: 1
- Safe: 1
- Signpost: 1
- Simple: 1
- Soil: 1
- Starting up: 1
- Straw based: 1
- Suitable: 1
- Untidy: 1
- Windswept: 1
- Woodland: 1
- Workable: 1
- Young: 1

38. What do your pigs really enjoy in life? (Open question, n=67, responses=187)

Codes:

- Freedom: 35
- Food: 29
- Rooting: 22
- Company: 21
- Wallowing: 14
- Weather: 11
- Petting: 8
- Natural life: 7
- Shelter: 7
- Space: 7
- Outdoors: 6
- Play: 6
- Sleep: 6
- Enrichment: 5
- Comfort: 4
- Grazing: 4
- Natural behaviour: 4
- Water: 4
- Trees: 3
- Foraging: 2
- Variety: 2
- Diversity: 1
- Health: 1
- Long life: 1
- Nesting: 1
- Stress free: 1

Freedom explainers:

- Roaming: 13
- Social group: 2

Food explainers:

- Fruits/Vegies: 6
- Quantity: 2

Company explainers:

- Humans: 9
- Pigs: 7

Weather explainers:

- Sun: 9
- Summer: 1

39. What do your pigs least enjoy in life? (Open question, n=61, responses=142)

Codes:

- Weather: 44
- Nutrition issues: 16
- Confinement: 15
- Change: 10
- Moving: 10
- Fences: 7
- Isolation: 7
- Slaughter day: 7
- Stress: 7
- Pain: 4
- Boredom: 3
- Hard ground: 3
- Poor health: 3
- Aggression: 2
- Maltreatment: 2
- Death: 1
- Dog: 1
- Flies: 1
- Grief: 1
- Handling: 1
- Lack of space: 1
- Mud: 1
- Vet: 1

Weather explainers:

- Wet/Rain: 16
- Cold: 14
- Hot: 11
- Wind: 2
- Winter: 1

Nutrition issues explainers:

- Hunger: 10
- Thirst: 2

Change explainer:

- Social group: 6

Fences explainers:

- Shocks: 4

Pain explainer:

- Procedure: 3

40. Would you like to add any other information about your farm, your animals, your management, your daily life? (e.g., fences, surrounding landscape, other species kept...) (Open question, n=30)

Answers can be categorised in 5 groups related to:

1. Equipment and practices at 33.3% (n=9): Use of "electric fence" sometimes "portable" that have to be "daily checked", need of "strong fencing with electrical fencing", performed "paddock rotation" with the "use of kiwitech", use of "the bigger paddock with the most landscape variety [so the pigs do not] attempt to break out", "use of moveable shelter", importance of the use of the adapted breed so avoid health issues.

2. Challenges at 25.9% (n=7): make the farm "economical viable", "low support" from de department of agriculture, "stress" about "where the next customer/market will be", effort and quality product not rewarded, "find a butcher", "stress" due to highway going through the farm, keep animal for tourism during Covid, deal with the wind

3. Benefits at 22.2% (n=6): "happy having horse near them", "Chicken and ducks kept in tandem to minimise parasite build up in the soils", "pigs […] work with me to prepare the ground for vegetable plots", "rewarding" and "enjoyment", meat quality

4. Plans at 7.4% (n=2): agroforestry, manage pigs so they follow the layers hens mobile system for tillage rotation

5. Wish to help in the project at 7.4% (n=2).

6. Believes at 3.7% (n=1): importance of "seeing more pig outdoor"
