# Supplementary material for: Pigs and pasture: Drivers and characteristics of outdoor systems on the island of Ireland
Source: PLoS One. 2026 Feb 3;21(2):e0341440. doi: 10.1371/journal.pone.0341440 (PMC12867241; doi:10.1371/journal.pone.0341440)
Supplement: Attachment — Submitted filename: Response to Reviewers.docx [file pone.0341440.s003.docx]

Journal Requirements:

When submitting your revision, we need you to address these additional requirements.

1. Please ensure that your manuscript meets PLOS ONE's style requirements, including those for file naming. The PLOS ONE style templates can be found at

<https://journals.plos.org/plosone/s/file?id=wjVg/PLOSOne_formatting_sample_main_body.pdf> and [https://journals.plos.org/plosone/s/file?id=ba62/PLOSOne_formatting_sample_title_authors_affiliations.pdf](https://track.editorialmanager.com/CL0/https:%2F%2Fjournals.plos.org%2Fplosone%2Fs%2Ffile%3Fid=ba62%2FPLOSOne_formatting_sample_title_authors_affiliations.pdf/1/010f0198371c7659-f8ea5466-f701-4da6-a24e-956addd48365-000000/TcB9CvdviboLpwJjdnYr7L0TvzIPGt9mKbEjcfA8yUg=221)

The new version of the manuscript follows the PLOS ONE’s style.

2. Please provide additional details regarding participant consent. In the ethics statement in the Methods and online submission information, please ensure that you have specified what type you obtained (for instance, written or verbal, and if verbal, how it was documented and witnessed). If your study included minors, state whether you obtained consent from parents or guardians. If the need for consent was waived by the ethics committee, please include this information.

Details regarding consent are included in lines 88-90 in the materials and methods section. The participants were asked to confirm consent by ticking a box at the beginning of the online survey. We have included the information that participants could not progress to the survey unless this box was ticked.

3. Thank you for stating the following financial disclosure:

[This study was funded by the Department of Agriculture, Food and the Marine in Ireland (Grant number 2021R600).].

Please state what role the funders took in the study.  If the funders had no role, please state: "The funders had no role in study design, data collection and analysis, decision to publish, or preparation of the manuscript."

If this statement is not correct you must amend it as needed.

Please include this amended Role of Funder statement in your cover letter; we will change the online submission form on your behalf.

The statement regarding the financial disclosure is in the cover letter.

4. Thank you for stating the following in your manuscript:

[This study was funded by the Department of Agriculture, Food and the Marine in Ireland (Grant number 2021R600).]

We note that you have provided funding information that is currently declared in your Funding Statement. However, funding information should not appear in the Acknowledgments section or other areas of your manuscript. We will only publish funding information present in the Funding Statement section of the online submission form.

Please remove any funding-related text from the manuscript and let us know how you would like to update your Funding Statement. Currently, your Funding Statement reads as follows:

[This study was funded by the Department of Agriculture, Food and the Marine in Ireland (Grant number 2021R600).]

Please include your amended statements within your cover letter; we will change the online submission form on your behalf.

The funding statement should appear as followed: “This study was funded by the Department of Agriculture, Food and the Marine in Ireland (Grant number 2021R600). The funders had no role in study design, data collection and analysis, decision to publish, or preparation of the manuscript.” The cover letter contains the request for this amendment.

5. When completing the data availability statement of the submission form, you indicated that you will make your data available on acceptance. We strongly recommend all authors decide on a data sharing plan before acceptance, as the process can be lengthy and hold up publication timelines. Please note that, though access restrictions are acceptable now, your entire data will need to be made freely accessible if your manuscript is accepted for publication. This policy applies to all data except where public deposition would breach compliance with the protocol approved by your research ethics board. If you are unable to adhere to our open data policy, please kindly revise your statement to explain your reasoning and we will seek the editor's input on an exemption. Please be assured that, once you have provided your new statement, the assessment of your exemption will not hold up the peer review process.

The data are now available at <https://opendata.teagasc.ie/dataset/survey_production_health_welfare_outdoor_pig_farms_ireland>.

If the reviewer comments include a recommendation to cite specific previously published works, please review and evaluate these publications to determine whether they are relevant and should be cited. There is no requirement to cite these works unless the editor has indicated otherwise. 

**Comments to the Author**
**Reviewer #1:** This study is appropriate for internal and epidemiological journals. This study is appropriate for internal and epidemiological journals, as it offers valuable insights into the patterns and determinants of disease spread within specific populations. By leveraging both quantitative and qualitative data, it provides a comprehensive overview of health trends that can inform preventive strategies and intervention policies.

We thank reviewer #1 for highlighting the valuable information about pig health raised on outdoor systems that our work has generated.

**Reviewer #2:** This paper provides a comprehensive review of outdoor pig farming systems in Ireland, a previously understudied area. Using an online questionnaire and analysis of qualitative and quantitative data, the authors were able to comprehensively identify farmers’ motivations, challenges, and needs in the sector. This study fills a gap in the literature on small-scale, decentralized pig farming systems and provides valuable insights for policymakers and industry practitioners.

We would like to thank the reviewer #2 for the thoughtful and constructive feedback on our work. We hope that our changes in the manuscript answer the reviewer’s questions.

I have some minor questions:
What factors have been identified as the main drivers for pig farming in open systems in Ireland and how do these relate to financial and infrastructural challenges?

The survey identified several reasons for pig keeping in outdoor pig farms, as well as some of the drivers as to why respondents keep pigs this way. The purpose for which respondents keeping pigs in outdoor farms is described in L173-178, and Figure 1. The reasons why they feel keeping pigs this way is important is described in the section from L323 – 327. These include animal welfare, meat quality, traceability and environmental sustainability.

We did not carry out an analysis to investigate whether there is a relationship between what respondents reported as being their main drivers for keeping pigs outdoors, and the answers to the questions about challenges and changes needed at infrastructural level. The main challenges identified are listed from L339 – 348, and Figure 5A, and the changes from L355-364. However, education was the change that is needed that was most commonly included, in relation to consumers, farmers, and regarding animal welfare and the environment. Challenges mainly related to finances. Thus, it is possible that respondents consider that improving education amongst consumers regarding the drivers for their production systems could help to support their businesses by resulting in more people purchasing their meat (L523-525). In the conclusion (Lines 558-560) we have also included the following statement: “Farmers see animal welfare, meat quality, and food provenance as drivers in raising pig outdoor and prioritized them, though challenges such as feed costs, financial support, and soil maintenance persist.”

Are there significant differences in pig health and welfare management between small and large farms in open systems in Ireland?

As described in the study, outdoor pig systems in Ireland are small scale (median 21 animals). We were unable to identify any large scale outdoor farming enterprises on the Island, and consequently no farm scale comparison on animal health could be performed. Authors could not find any study performed in other countries that compare small and large outdoor farms to rate the pig health reported in their study and former ones.

What is the role of traditional and rare pig breeds in the success of open systems in Ireland and how are these breeds adapting to climate conditions and market demands?

The result of this study does not allow making a direct link between the choice of raising traditional pig breeds and the success/sustainability of outdoor systems in Ireland. This interesting question should be further studied, so our conclusion has been modified to “Future research should focus on breed-specific adaptation to outdoor conditions, meat quality and market demand, and environmental management strategies to support outdoor pig farming in Ireland, while ensuring economic viability and high welfare standards.” (Line 563-566)

What solutions have been proposed to improve the economic and environmental sustainability of pig farming systems in Ireland?

This study comprised the first time ever that there has been a nationally funded research project looking at small/outdoor pig production – to date there has not even been a national advisory or certification programme for this sector, to support farmers. In fact, this is one of the reasons that the project was funded by the Department of Food, Agriculture, and the Marine (DAFM), to obtain information about how this sector is structured, and the challenges that it faces, to help identify strategies to support it. As part of the wider project we have included a task to develop an industry led roadmap towards higher welfare pig production on the island of Ireland. This is led by Prof. Siobhan Mullan, who is one of the authors of this paper. The group includes stakeholders from across the entire pig sector, including outdoor farmers, and representatives from DAFM. The aim is to identify strategies as you suggest, to help improve the sustainability of the sector, while hopefully improving welfare standards, and set aims and targets that could be met in 5, 10, 20 years etc. The project is still ongoing, but the final report and roadmap that we produce from this and other tasks will comprise the first step in suggesting solutions nationally. The Irish Animal Welfare Strategy (2021-2025) incorporates the guiding principles of working in partnership, science-based policymaking, improving education, consistent evaluation, and an effective regulatory system, and as such the project feeds into this in a structured fashion. Our ambition is that our outputs will help to guide policies to support continuation and expansion of higher welfare, and outdoor pig production in Ireland.

**Reviewer #3:** "Pigs and Pasture: Drivers and characteristics of outdoor systems on the island of Ireland"
The study addresses a significant gap in the literature by characterizing outdoor pig production systems on the island of Ireland, a topic previously under-researched compared to intensive indoor systems. The focus on small-scale, outdoor systems provides novel insights into farmer motivations, management practices, and challenges, contributing to broader discussions on sustainable agriculture, animal welfare, and alternative farming models. The inclusion of both commercial and non-commercial producers adds depth to the analysis.

We would like to thank the review #3 highlighting the relevance of our study and the variety of topics addressed in it. We hope that the changes made in the manuscript clarified the reviewer’s concerns about the data collection and analyses.

However, concerns and issues requiring revision should be addressed:
Sample Representativeness and Bias: The study relies on self-selected respondents (n=90), which may overrepresent farmers engaged in welfare-focused or organic production, skewing results. No response rate is calculable due to the unknown total population of outdoor pig farms, limiting generalizability.

As the reviewer indicated, our study is based on voluntary participations that might bias results; however, participants were not selected based on their engagement on animal welfare nor organic production. As the study focused on outdoor pig farm systems the selection criteria was that respondents had to raise pigs outdoors as stipulated lines 127-130. Reasons for not including responses in the analyses were explained lines 130-131. We have acknowledged that it is not possible to estimate response rate at the beginning of the discussion, L404 - 406.

Health data reliability: Self-reported health metrics (e.g., only 31% reporting health issues) conflict with field studies cited (e.g., 80% parasite prevalence in a subset of farms). This suggests underreporting or lack of farmer expertise in disease recognition. No objective health assessments (e.g., veterinary records, lab tests) were used, risking bias.
Recommended Revisions: Thus, address health data discrepancies by cross-referencing self-reports with veterinary records or farm visits.

The reviewer highlighted a common bias of survey studies, which is the subjectivity, and/or awareness of the participants when answering. We have changed the wording in L461 to reflect that the lack of evidence many health issues was based only upon survey results. This bias is also discussed from lines 467-472. In brief, following on from this survey, we carried out a study whereby we visited a sub-set of farms to assess the pigs’ health and welfare. Ultimately this will enable us to compare the differences in results between the self-reported data in the survey, and the field work. To date, we have published the first paper from this work, on the subject of gastrointestinal parasites (Senanayake et al., 2025), and found that gastrointestinal problems may be under-reported. More data collected during the field work (e.g. body condition, lameness, lesion scores etc.) has been analysed, and a manuscript is currently in preparation. These animal based measures will be compared and discussed in the context of the survey results in that paper.

Reference:

Senanayake, S.N., Boyle, L., O’Driscoll, K., Menant, O. and Butler, F. 2025. Effects of season, age and parasite management practices on gastro – intestinal parasites in pigs kept of outdoors pig farms in Ireland. Irish Veterinary Journal. 78:12 <https://doi.org/10.1186/s13620-025-00297-0>

Methodological gaps: Open-ended question coding lacks inter-rater reliability metrics, raising questions about consistency in qualitative analysis. Co-occurrence analysis, while useful, does not account for confounding variables (e.g., farm size influencing challenges like feed costs).
Recommended Revisions: Clarify qualitative coding methodology (e.g., inter-rater agreement).

More details about the answers format of the open questions analysed, and the coding methodology were added in the Materials and methods section of the manuscript in L108-109, and L139-148.

In the analysis section the sentence “Due to the diversity in farm systems, no farm characteristics were included as confounding variables in the co-occurrence analyses.” was added to explicitly inform that no confounding variables were used for the co-occurrence analysis (L161 – 163).

Geographic and comparative limitations. Findings are specific to Ireland’s small-scale systems; comparisons to larger EU outdoor systems (e.g., UK, Spain) are minimal, reducing broader applicability. Climate/soil differences (e.g., Ireland’s high rainfall vs. Mediterranean systems) are noted but not deeply analyzed for their impact on challenges like paddock management.
Recommended Revisions: Expand comparative discussion to EU outdoor systems.

As highlighted by the reviewer, the wide diversity of outdoor farms systems and climate conditions across Europe make comparison between systems challenging. We did not identify any large-scale outdoor pig farming operations that are comparable to the systems that are commonly found in for example the UK, or Spain. For this reason, we were unable to include a comparative discussion between the systems in our findings and those observed in Europe. We have noted this in L414-415. (Thus in-depth comparisons with these systems are not relevant in the context of outdoor Irish pig production).

We also added at the end of the discussion, L 553-554, “In Mediterranean regions, although the rainfall is lower, soil erosion is also a challenge on sandy-loam farm with high animal stocking density (51).”

Ambiguity in definitions: Organic/GM-free standard" is self-reported without verification of certification, potentially misclassifying farms. "Extreme weather" is not quantitatively defined (e.g., temperature thresholds, rainfall levels), making it subjective.
Recommended Revisions: Define key terms (e.g., "extreme weather") operationally.

The reviewer is right, the delineation of some categories and the definition of some word or expression are not well described. However, we cannot provide additional detail over the way these are described in the manuscript because this is the wording that was used in the survey, so we are simply reporting the answers provided by the participants. We did not provide explanatory information in the survey, which is something that we acknowledge could be done in future surveys. To clarify, we have added “multiple-choice question” to the text in L96-97 to clarify how the question was asked.

We have changed the term ‘extreme weather’ to ‘inclement weather’, as this describes more clearly that we mean weather that could be uncomfortable for the animals (L38 and 71).

Policy and financial analysis superficiality: Calls for "financial support" and "consumer education" lack specificity (e.g., proposed subsidy structures, educational campaigns). No cost-benefit analysis of suggested interventions (e.g., mobile abattoirs) is provided.
Recommended Revisions: Propose concrete policy measures (e.g., grant schemes, training programs).

Proposing of concrete policy measures is beyond the scope of the current study. Participants were asked to answer the open questions only using one word or expression (information added lines 108-109), so it is not possible to extrapolate potential policy measures that they suggest might be useful from this. Furthermore no specific details about finances were asked, so it is also unfortunately, impossible to provide a cost-benefit analyse nor give details about which type of schemes and programs outdoor pigs farmers would like to benefit from. However, this survey is part of a wider project that will help to answer some of these questions. As stated in the answer to another reviewer, the project includes a task to develop a roadmap towards higher welfare pig production in Ireland, which will include these suggestions. The project also includes a task looking at economic and social life cycle assessment of higher welfare and outdoor pig farming systems. Finally, there is also a dedicated social science task, where these questions are also being explored. The aim of the project is to publish peer-reviewed papers in all these areas, and we consider it appropriate to wait for these more objective studies rather than propose policy measures in the current manuscript.

Data availability timing Data will be available post-publication, hindering immediate reproducibility. A preprint or supplemental upload at submission would strengthen transparency.

Data are now available at <https://opendata.teagasc.ie/dataset/survey_production_health_welfare_outdoor_pig_farms_ireland>.

Recommended Revisions: These revisions would enhance rigor and applicability without altering the study’s core contributions.

We would like to thank the reviewer #3 for all these interesting comments that helped us improving this work.
